# Supplementary material for: Individuals with autism spectrum disorder have altered visual encoding capacity
Source: PLoS Biol. 2021 May 12;19(5):e3001215. doi: 10.1371/journal.pbio.3001215 (PMC8143398; doi:10.1371/journal.pbio.3001215)
Supplement: S1 Text — (DOCX) [file pbio.3001215.s001.docx]

**S1 Text**

Perceptual inference is often described as an encoding-decoding process. Sensory stimuli, $\theta,$ are first *encoded* by a noisy neural measure, $p\left( m | \theta\right)$ - i.e., objects we wish to encode are mapped onto a noisy and limited neural representation. Second, these measurements, $m$, inform an observer about the likely sensory surrounding, and the latter is combined with *prior* expectations about the environment, $p\left( \theta\right)$, in forming a posterior belief $p\left( \theta| m \right)$. Lastly, an estimate of the stimulus, $\hat{\theta}(m)$ is generated based on the posterior and the *loss function* subjects employ for the task. The latter two stages represent the “decoding” stage. The general nature of this formulation allows for attribution of behavioral variations to different components within the framework (i.e., encoding, prior, and loss function; **Figure 1**). Previous research has shown that these components can indeed be quantitatively “reverse engineered” from psychophysical data [R1, R2], which serves as the basis for the comparison across individuals and groups [54]. However, there are several important limitations in the “reverse-engineering” approach employed in previous ASD studies. To fit the encoding-decoding model to psychophysical data, the form of conditional probability (which determines the likelihood function) and prior are usually assumed to be within a restrictive parametric family, and the loss function is often assumed to be simple and identical across subjects (e.g., Least Square Error,$L_{2}$, loss, but see [R3]). This is problematic for the following reasons:

First, the repulsive bias pattern in orientation estimation turns out to be challenging to model with the traditional Bayesian framework, and a more principled encoding scheme (i.e., efficient coding, which suggests that encoding is also directly constrained by the prior) has been proposed to explain the observed effect [23]. Namely, it is exactly the properties of encoding that leads to the observed perceptual bias.

Second, we are interested in sources of variation that may be multifaceted across subjects, groups (neurotypical vs. ASD), and feedback sessions in our experiment. It is not clear *a priori* what particular assumptions of the parametric family of distributions and loss functions are suitable for an unbiased estimation across all three components of the framework.

Third, previous research showed that factors beyond early perception may also contribute to repulsive biases in orientation estimation (e.g., post-decision bias, [52]). A similar argument applies to putative perceptual learning within the current experiment. Namely, subjects may develop complex and idiosyncratic strategies to reduce error across feedback sessions. While these effects can potentially be accounted for within a Bayesian model, more complicated forms of prior may be required (e.g., [52, R4]).

Instead, we employed a simple, yet powerful, method for data analysis that only focusses on the sensory encoding stage, aiming to selectively examine the capacity and flexibility of sensory representation in individuals with ASD, free of any parametric *a priori* assumptions, while remaining *agnostic* about the decoding process. We utilize the Cramer-Rao Lower Bound [15, 16], which states that the bias and variance produced by *any* estimator obeys the following relationship:

$$\text{var}\left( \hat{\theta} \right)\geq\frac{\left[ 1+b^{'}\left( \theta\right) \right]^{2}}{I_{F}\left( \theta\right)}$$

Here $b\left( \theta\right)$ denotes the bias of the estimator and $I_{F}\left( \theta\right)$ denotes Fisher Information (FI), both expressed as a function of the stimulus, $\theta$. Rearranging the equation, we have $I_{F}\left( \theta\right)\geq\frac{\left[ 1+b'\left( \theta\right) \right]^{2}}{\text{var}\left( \hat{\theta} \right)}$. Thus, we can extract a lower bound on the FI based on the bias and variance of subjects’ responses. Furthermore, when the bound is tight (i.e., $\geq$ approximates =), we have a direct estimate of the FI of subjects’ encoding. Note that the assumption that the bound is tight is rather minimal, given that maximum likelihood and Bayesian estimators, both widely used decoders in the literature, typically attain the Cramer-Rao Lower Bound for simple estimation problems. Below we show analytically this is indeed the case for a Gaussian likelihood/Gaussian prior problem with *arbitrary* parameters. Together with the numerical simulation we have presented (**Figure 1**), we demonstrated that our method allows to directly estimate the encoding characteristics in terms of Fisher Information, while remain agnostic and flexible about the (potentially diverse and idiosyncratic) decoding process employed by each subject. Further, under the efficient coding hypothesis that encoding is determined by the stimulus statistics, we can then infer subjects’ prior distribution from the characteristics of their encoding.

*Analytical Derivation*

We are interested in estimating the stimulus value $\theta$ based on the sensory measurement $m$. We assume the encoding can be described as a Gaussian conditional probability:

$$p\left( m | \theta\right)\mathcal{=N}\left( m;\theta,\sigma_{s}^{2} \right)$$

The Fisher Information of the encoding is a constant:

$$I_{F}\left( \theta\right)=1/\sigma_{s}^{2}$$

*Decoder 1: Maximum Likelihood Estimator*

The maximum likelihood estimator (MLE) for our simple encoding model is$\hat{\theta}_{MLE}(m)=m.$ Thus, $p\left( \hat{\theta} | \theta\right)\mathcal{=N}\left( \hat{\theta};\theta,\sigma_{s}^{2} \right)$, and we have $E\left( \hat{\theta} | \theta\right)=\theta, \text{b}\left( \theta\right)=0,$ and $\text{var}\left( \hat{\theta} | \theta\right)=\sigma_{s}^{2}$. We can see that the MLE attains the Cramer-Rao lower bound since:

$$\sigma_{s}^{2}=\frac{\left[ 1+b'\left( \theta\right) \right]^{2}}{I_{F}\left( \theta\right)}=\frac{1}{1/\sigma_{s}^{2}}$$

*Decoder 2: Arbitrary Combinations of Gaussian Prior and Gaussian Likelihood*

We can construct a Bayesian decoder by assigning a Gaussian prior distribution on $\theta:p\left( \theta\right)\mathcal{=N}\left( \theta;\mu_{0},\sigma_{0}^{2} \right).$ The posterior $p\left( \theta| m \right)$ is of the form:

$$p\left( \theta| m \right)\mathcal{=N}\left( \theta;\frac{\tau_{s}}{\tau_{s}+\tau_{0}}m+\frac{\tau_{0}}{\tau_{s}+\tau_{0}}\mu_{0}, \frac{1}{\tau_{s}+\tau_{0}} \right)$$

where $\tau_{s}=1/\sigma_{s}^{2}$, and $\tau_{0}=1/\sigma_{0}^{2}$. Due to the symmetrical nature of the posterior, estimators $\hat{\theta}_{\text{bayes}}$ correspond to loss functions $L_{0},L_{1}$ and $L_{2}$ norm are of the same form, namely posterior mean:

$$\hat{\theta}_{\text{bayes}}(m)=\frac{\tau_{s}}{\tau_{s}+\tau_{0}}m+\frac{\tau_{0}}{\tau_{s}+\tau_{0}}\mu_{0}$$

Denote $\frac{\tau_{s}}{\tau_{s}+\tau_{0}}=k$, and $\frac{\tau_{0}}{\tau_{s}+\tau_{0}}\mu_{0}=c_{0}.$ According to linear transformation of Gaussian random variable, we can show that $p\left( \hat{\theta} | \theta\right)\mathcal{=N}\left( \hat{\theta};k\theta+c_{0},k^{2}\sigma_{s}^{2} \right).$ Thus, $E\left( \hat{\theta} | \theta\right)=k\theta+c_{0}, \text{b}\left( \theta\right)=\left( k-1 \right)\theta+c_{0},$ and $\text{var}\left( \hat{\theta} | \theta\right)=k^{2}\sigma_{s}^{2}.$ Again, we can show that $\hat{\theta}_{\text{bayes}}$ is at the Cramer-Rao lower bound for arbitrary parameters of the prior mean $\mu_{0}$ and variance $\sigma_{0}^{2}$:

$$k^{2}\sigma_{s}^{2}=\frac{\left[ 1+b'\left( \theta\right) \right]^{2}}{I_{F}\left( \theta\right)}=\frac{\left( 1+k-1 \right)^{2}}{1/\sigma_{s}^{2}}$$

To conclude, for the same encoding process with uniform Fisher Information $I_{F}\left( \theta\right)=1/\sigma_{s}^{2}$, we can construct unbiased MLE and Bayesian estimators with an arbitrary prior and thus an arbitrary bias. Importantly, both of these estimators, by far the most used in neuroscience, attain the Cramer-Rao lower bound. Furthermore, notice that for the Bayesian estimator we have $\text{var}\left( \hat{\theta} | \theta\right)=k^{2}\sigma_{s}^{2}$, where $k\leq1$. This indicates that the introduction of the prior, while causing a bias, reduces the variance in the estimate (i.e., there is an inherent give and take between bias and variance, as indicated by the Cramer-Rao bound).

**Supplementary References**

[R1] Stocker A.A., Simoncelli E.P. (2006a). Noise characteristics and prior expectations in human visual speed perception. Nat. Neurosci. 9:578–85

[R2] Kording K.P., Wolpert DM. 2004. Bayesian integration in sensorimotor learning. Nature 427:244–47

[R3] Bates, C. J., Lerch, R. A., Sims, C. R., & Jacobs, R. A. (2019). Adaptive allocation of human visual working memory capacity during statistical and categorical learning. Journal of vision, 19(2), 11-11.

[R4] Qiu, C., Luu, L., & Stocker, A. A. (2020). Benefits of commitment in hierarchical inference. Psychological Review, 127(4), 622.
