# Supplementary figures and images for: Individuals with autism spectrum disorder have altered visual encoding capacity
Source: PLoS Biol. 2021 May 12;19(5):e3001215. doi: 10.1371/journal.pbio.3001215 (PMC8143398; doi:10.1371/journal.pbio.3001215)

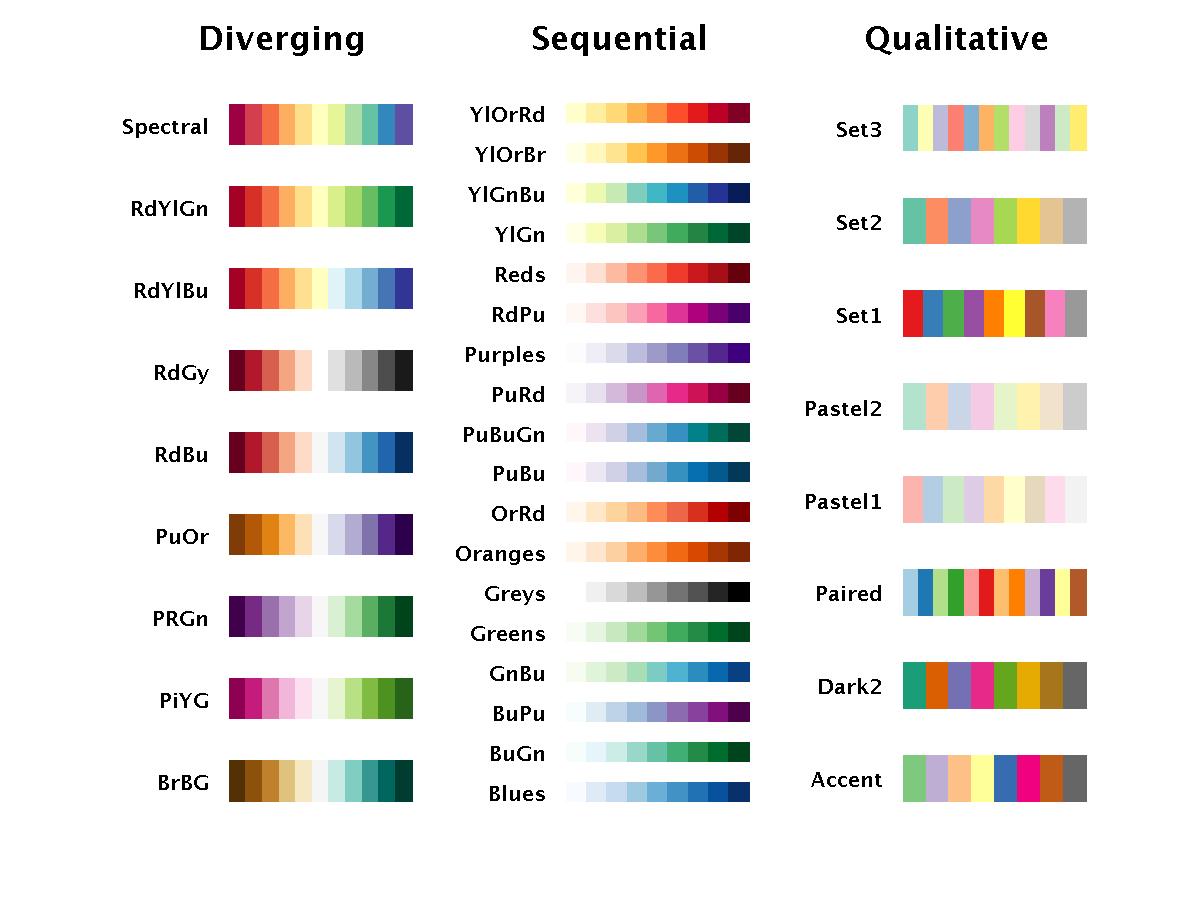

Supplement: S1 Code — Routine to reproduce Figs 1D–1F, 2B, 2C, 3, 4 and 5 and S1–S6, panel B in S7, and S8 Figs. (ZIP) [file pbio.3001215.s010.zip › ASD_Encoding_2020-release/cbrewer/cbrewer_preview.jpg]
