# Supplementary material for: Individuals with autism spectrum disorder have altered visual encoding capacity
Source: PLoS Biol. 2021 May 12;19(5):e3001215. doi: 10.1371/journal.pbio.3001215 (PMC8143398; doi:10.1371/journal.pbio.3001215)
Supplement: Attachment — Submitted filename: Review.pdf [file pbio.3001215.s017.pdf]

This study investigates sensory encoding in autism (ASD). Over the past decade, there has been an increase in work looking at sensory abnormalities in autism, but we still lack a good understanding of how sensory, namely visual, processing is atypical in autism. This study focuses on visual encoding, a critical early step in visual processing. This question is addressed in the context of visual orientation discrimination and combines convincing behavioral data (I'd say remarkably convincing given what is typically seen in ASD work) and sophisticated computational modeling. The conclusion is that sensory encoding is severely impaired in ASD.

I find this to be an interesting and a noteworthy study. The behavioral data are, to repeat, convincing and certainly indicate atypical orientation processing in ASD. The modeling is well done, and while I'm not ready to fully "buy" the ultimate conclusions (details below), even if those conclusions turn out to be not entirely correct, this work will still be an important addition to our understanding of how sensory processing could be impaired in ASD.

1A. There are two key behavioral findings: (1) the overall difference in performance in the first block and (2) poor learning/adaptation of the ASD group over blocks. Both findings need to be better integrated with the existing literature, both to better contextualize the present results and to consider alternative explanations. The authors do link the first result to other studies showing increased variability in ASD, but more is needed there. For example, a recent study that examined variability of visual orientation processing is not mentioned. Also, it is worth mentioning work that questions the "noise hypothesis" in ASD. Both references are below.

> Park, W. J., Schauder, K. B., Zhang, R., Bennetto, L., & Tadin, D. (2017). High internal noise and poor external noise filtering characterize perception in autism spectrum disorder. *Scientific reports*, *7*(1), 1-12.
>
> Butler, J. S., Molholm, S., Andrade, G. N., & Foxe, J. J. (2017). An examination of the neural unreliability thesis of autism. *Cerebral cortex*, *27*(1), 185-200.

1B. In addition, the change in performance over blocks is discussed as learning, but no work on perceptual learning in ASD is mentioned. For example, the paper listed below showed that perceptual learning in ASD is atypical. This raises the question of whether the present results could be a result of a boarder deficit in learning?

> Harris, H., Israeli, D., Minshew, N., Bonneh, Y., Heeger, D. J., Behrmann, M., & Sagi, D. (2015). Perceptual learning in autism: over-specificity and possible remedies. *Nature neuroscience*, *18*(11), 1574-1576.

2. The first block does not use feedback, while the 2nd and the 3rd blocks do. That decision needs to be explained and justified, but it is not. Why was adding feedback after the first block important? After all, feedback is not necessary for us to learn stimulus statistics (though it likely speeds up the process).

My worry is that this leaves open a possibility that how the two groups responded to this change in feedback could be, in part, what drives the difference. For example, what if individuals with ASD ignored feedback? (btw, this is a hypothetical, I don't have a reason to believe this). What I'm looking for here is a better justification of why the feedback was used in this specific way and are there good reasons to believe that the results would generalize to other ways of deploying feedback (or no feedback at all). That is, I'm not asking for new data (certainly not during COVID times).

3. The AQ results (Fig 5) are entirely un-informative. The reported correlations seem to be mostly driven by the overall group differences, with little evidence of within-group correlation effects. For these conclusions to stand, what is needed is evidence that **within** the ASD group, these results are corelates with the symptom severity.

Moreover, AQ is a somewhat outdated measure that relies a lot on imperfect stereotypes of what autism is, and it is not used in the diagnosis process (rather it is best used as a measure of autism-like behaviors and thinking among the general population). However, it does look like ADOS was collected.

Minor points:

-- I'd take out "patients" from the title. Many individuals with autism do not consider themselves to be patients.

-- In discussion (page 9), it is stated "This speculation will have to be confirmed in physiological studies." It is not clear how. Are we talking about EEG and fMRI or animal models?

-- Materials and Procedures: stimulus sizes need to be given in visual degrees and not centimeters.
