# Supplementary material for: Individuals with autism spectrum disorder have altered visual encoding capacity
Source: PLoS Biol. 2021 May 12;19(5):e3001215. doi: 10.1371/journal.pbio.3001215 (PMC8143398; doi:10.1371/journal.pbio.3001215)
Supplement: Attachment — Submitted filename: PlosBio_Letter_to_reviewers.docx [file pbio.3001215.s018.docx]

**Reviewer #1:**

The authors use the oblique effect to examine sensory encoding in neurotypical and autistic individuals. They report that after undergoing trials with feedback, controls, but not autistic participants change their sensory representations and 'increase encoding capacity'. This, they conclude, suggests deficits in sensory encoding in autism and compromised ability to adapt to novel input statistics. I find the notion of autism being associated with inflexible priors quite appealing, given its potential explanatory scope for many facets of the phenotype. However, I am not persuaded that the empirical evidence presented here provides unequivocal support for this proposal. My concerns are related to the experimental paradigm used and also the interpretation of some of the findings.

1. The oblique effect strikes me as a less than ideal phenomenon to use for probing dynamic changes in priors. Although the neural basis of this effect is not definitively established, studies that have examined responses from large collections of neurons in visual cortex suggest that anisotropies in cell preferences as early as V1 may largely be responsible for the phenomenon (e.g. Baowang Li, Matthew R. Peterson, and Ralph D. Freeman (2003). Oblique Effect: A Neural Basis in the Visual Cortex. J Neurophysiol 90: 204-217). Response properties of neurons in early sensory cortex, that have been shaped by long evolutionary history, are not readily modifiable by short experiences. Hence, the oblique effect may not be the ideal vehicle to assess the impact of short-term changes in image statistics on sensory encoding.

We thank the reviewer for this interesting observation and for highlighting the very relevant paper he or she points to. We examined the oblique effect, because it is broadly thought to reflect aspects of sensory encoding, as opposed to decoding - which has been the emphasis in the computational study of Autism. Indeed, it is very likely that the statistics of our environment guide the distribution of tuning functions in early sensory areas, such as V1, and that these tuning functions play an important role in the oblique effect. However, an array of other elements - e.g., noise correlations, neuromodulators (gain modulation), etc. - impact how environmental energies are mapped onto bandwidth limited neural responses. Indeed, the fact that we find a large impact of feedback on the total encoding capacity and allocation thereof in the control population is testament to the fact that the oblique effect and our protocols were capable of indexing a dynamic change in encoding. Related, prior studies on perceptual learning have also shown rapid changes in encoding in terms of discrimination threshold, including encoding of orientations (Vogels & Orban, 1985; Shiu & Pashler, 1992). Similarly, the spatial and temporal context of the stimulus (i.e., sensory adaption) can also influence the response properties of early sensory neuron (including the shape of its tuning function), sometimes on an even shorter timescale than our current experiment (Clifford et al., 2007; Schwartz, Hsu & Dayan, 2007; Solomon & Kohn, 2014). We have amended the *Introduction* to further motive the use of our experimental protocol:

*To identify anomalies in the capacity, and the updating of sensory encoding in ASD, we took advantage of the well-known “oblique effect” [17, 18]. That is, humans demonstrate greater sensitivity and repulsive (i.e., away from) biases in perceiving gratings and motion around cardinal compared to oblique orientations [19, 20]. This effect is thought to reflect the fine tuning of sensory encoding to environmental statistics – with vertical and horizontal orientations being most common in nature [21, 22]. Recent studies [16, 23] further highlight that it is exactly this anisotropic property of sensory encoding that leads to the observed repulsive bias. In other words, the oblique effect is thought to reflect a property of sensory encoding, and not decoding. A standard Bayesian decoder with homogenous encoding cannot account for the oblique effect, given the bias is away from and not toward the prior [23]. Lastly, early studies demonstrated that orientation discrimination is readily impacted by perceptual learning, adaptation, and feedback (e.g., [24, 25, 26]) on a relatively short time-scale. In turn, orientation estimation is an appropriate paradigm to study the updating and flexibility of the encoding process.*

We have also amended the *Discussion* to describe more broadly potential neural mechanisms driving the effects we report. These sections read as follows:

*There is a wide range of potential neural mechanisms by which the amount and allocation of encoding resources may change on a timescale that is compatible with the duration of our experiment. While neural recordings in animal models have shown that practicing orientation discrimination can reshape tuning functions as early as V1 [43], given the timeframe of the current experiment, we consider that network-level mechanisms are most likely. A first possibility is a gain modulation of the neural response. Indeed, for an efficient coding framework where each cell transmits an equal portion of the stimulus probability mass, an increase in the overall firing rate corresponds to a direct increase in population FI [44]. Previous studies of sensory adaption (e.g., [26, 45]) have also suggested that gain changes specific to a subpopulation of neurons are able to alter the allocation of coding resources. Gain modulation could be implemented by alterations in neuromodulation, and fittingly Lawson and colleagues, 2017 [10], recently demonstrated abnormal noradrenergic responsivity in ASD and suggested that this anomaly is related to the slow updating of their priors. The present findings suggest a functional interpretation for such a link: abnormal noradrenergic responsivity could decrease FI (e.g., encoding resources), which then directly correlates with an inflexibility in altering priors. A second potential mechanism may be changes in the noise-correlation structure in the neural population. For example, a decrease in inter-neuronal correlations generally reduces the impact of noise on stimulus representation, thus leading to a higher population FI [46]. Along this line, Coen-Gagli & Solomon, 2019 [47], have recently suggested that divisive normalization [48] is a critical player in neural variability, and Rosenberg and colleagues, 2015 [49], have accounted for a wide array of perceptual deficits in ASD by postulating a deficit in divisive normalization.*

2. The task that participants are asked to perform relies not just upon encoding, but also short-term memory (to remember the seen orientation until a response is made) and the ability to avoid 'contamination' of the remembered orientation by the perceived orientation of the response Gabor itself. In this context, can the differences in performance between controls and ASD participants be unambiguously ascribed to encoding differences alone? It is possible that ASD participants are more susceptible to contamination between response probe orientation and remembered orientation, and hence do not show the kinds of gains in performance that controls do across the experimental blocks. (I might have missed this in the manuscript, but it is important to state how long it takes subjects in the two groups to respond, i.e. reorient the probe Gabor from its random starting orientation to the final one. How monotonic is this process? Do subjects exhibit overshoots and corrections? Do the different groups differ in this? Might ASD participants be more or less impulsive in their responses relative to controls and might this impact their response bias? All of this is to make the point that the process of responding may be somewhat protracted and involve inadvertent modifications of the remembered target orientation.)

We thank the reviewer for raising these interesting points. Of course, making a response in this task or *any* other visual psychophysics will require an element of short-term memory and motor response. However, we want to emphasize that one of the main contributions of our current work is in applying a particular data analysis methodology itself, that is, the Cramer-Rao bound, to the study of ASD. This method will appropriately index sensory encoding, even if there are differences in these elements, as long as they do not introduce *stimulus-dependent noise*. Thus, one would have to conjecture a reason why differences in the impact of “contamination” across control and ASD are orientation-dependent, but *not* part of the encoding process, to suggest that these elements confound the current results. We explicitly mention the assumption regarding *stimulus-dependent noise* in the *Introduction*, and the fact that virtually all perceptual decoding models make this assumption as well. Further, to make this point clearer, we have moved what was **Figure S1** in the original submission to the main text (part of **Figure 1** now). This figure shows a simulation demonstrating that Cramer-Rao indeed recuperates sensory encoding, even in the face of other differences.

Introduction:

*In other words, by measuring bias (*$b)$ *and variance (*$\sigma^{2})$ *in estimates* $\left( \hat{\theta} \right)$ *of* $\theta$*, we can determine a lower bound of FI, i.e., the accuracy with which the stimulus variable is represented. Equating this bound with a direct index of sensory encoding (i.e., in effect replacing the greater than or equal sign with an equality in* ***Eq. 1****) requires a single assumption - that the translation from sensory representations to behavioral outputs was uncorrupted by stimulus-dependent noise. This assumption is made by virtually all decoders in perceptual science (e.g., Bayesian decoders and MLE). The exact form of the decoder (i.e., idiosyncrasies in the mapping from sensory estimates to motor outputs) does not matter beyond this single assumption, given that a decoder will trade off accuracy and variance. We further motivate and demonstrate the validity of our approach via both an analytical derivation (see* ***Supplementary Note****) and numerical simulation (****Figure 1)*** *showing that: 1) the same encoding process (****Figure 1D****) can produce very distinct patterns of estimation bias and variance given different decoders (****Figure 1E****), yet critically, 2) with the Cramer-Rao Lower Bound, we are able to precisely recover the encoding scheme in terms of FI, regardless of the differences in decoding (****Figure 1F****).*

Figure 1:

***Figure 1. Theoretical and Conceptual Framework. A)*** *Perception can be described as an encoding-decoding process. Stimulus is first encoded in the noisy and resource limited sensory representation* $m$*. An estimate* $\hat{\theta}$ *is generated given* $m$ *based on the decoding process. As an experimenter, we can only measure overt responses; characterized as a subject’s bias (*$\mu\left( \hat{\theta} \right)- \theta$*; difference between the average estimate and real stimulus value) and variance* $\sigma^{2}\left( \theta\right)$ *in their responses.* ***B)*** *The fidelity of encoding in healthy subjects is generally anisotropic due to uneven allocation of sensory resources determined by environmental statistics. For example, as shown here, the non-linear transformation between stimulus values and a neural space (solid red line) with homogenous noise results in higher uncertainty for orientations around the oblique than those around the cardinal. This can be characterized by a non-uniform profile of Fisher information* $I_{F}\left( \theta\right)$ *(solid green line). In our experiment, we imposed an artificial, uniform distribution of orientations, different from that of natural environment, which allows us to study whether and how both groups update their encoding under changing stimulus statistics.* ***C)*** *Cramer-Rao Lower Bound specifies the lawful relationship between bias* $b\left( \theta\right)$*, variance* $\sigma^{2}\left( \theta\right)$*, and encoding accuracy, i.e., Fisher Information* $I_{F}\left( \theta\right)$*, regardless of the decoding scheme. This allows us to directly characterize encoding, while remaining agnostic about the details of the decoding process (also see Supplementary Text).* ***D)*** *To demonstrate validity of our approach, we simulate an observer with anisotropic encoding process,* $p\left( m | \theta\right)$*, with a peak FI at 90°.* ***E)*** *As an example, we construct three arbitrary decoders (red, black, and blue), yielding very distinct pattern of estimation biases and variances, yet they all attain the Cramer-Rao Lower Bound.* ***F)*** *Applying the inequality, we estimate the (lower bound of) FI, which appropriately recovers the identical, true underlying pattern of FI in the encoding regardless of idiosyncrasies in the decoding process.*

Additionally, as suggested by the reviewer we have examined the response time data (these are included below, and as **Figure S5** in the supplement of the manuscript). Overall, each response takes approximately 3 to 4 seconds. The overall shape of the distributions are similar across groups, suggesting similar task strategies. There is a small yet significant difference in the median of the two groups, with the clinical group being slower. This effect is on the order of 150 ms, which is negligible vis-a-vis the duration of each trial and is suggestive of a reaction time difference, as opposed to a difference in task-strategy. More importantly, the fact that individuals with ASD show *both* a reduced FI and are slightly slower than the controls demonstrates that the reported effect is not a consequence of speed-accuracy trade-offs or differences in task strategy (which presumably would benefit speed and thus why encoding accuracy and precision is worse in ASD). Lastly, both the control and ASD group become faster in responding from woFB to wFB1 and ultimately wFB2, despite the fact that the encoding capacity remains *unchanged* in ASD group, again suggesting that differences in task strategy or putative differences in working memory is unlikely to account for the differing accuracy and precision in visual orientation estimation across ASD and control.

We have added mention to the response time data in the text in the following manner:

*Examination of the fitted λ values confirmed that at the outset of the experiment the ASD group had a lower total amount of FI compared to the control group (control vs. ASD at woFB; Δ = 4.352 ± 0.473, p < 10^-3^;* ***Figure 4D****). This effect is unlikely due to differences in task strategies, such as impulsivity or speed-accuracy trade-offs, given that the distribution of response times (RTs) was qualitatively similar across both groups, and if anything, slower in ASD, making this latter group deficitary in both speed and accuracy, and thus the effect not being one of speed-accuracy trade-offs (****Figure S5****). Over the course of the experiment, λ increased further for the control group (woFB vs. wFB2 in control: Δ = 2.094 ± 0.580, p < 10^-3^;* ***Figure 4D)*** *but this was not the case for the ASD group (Δ = -0.150 ± 0.441, p = 0.732,* ***Figure 4D****). These results suggest a lower overall encoding capacity in ASD than controls, and further a capacity that does not improve over the course of repeated feedback. We observed a significant decrease in median RT between woFB and wFB2 for both the control (ΔRT = -0.61 ± 0.069 sec, p < 10^-3^) and ASD group (ΔRT = -0.75 ± 0.114 sec, p < 10^-3^; see* ***Figure S5****), again suggesting that differences in task strategy or putative differences in working memory are unlikely to account for the differing accuracy and precision in visual orientation estimation across ASD and control.*

And:

*Second, one may wonder about the potential impact of additional stimulus-independent variability introduced by factors following encoding – such as an inefficient decoder (i.e., a decoder that produces larger variance than that predicted by the Cramer-Rao lower bound), or post-perceptual factors such as motor noise. We attempted to experimentally minimize the potential impact of motor noise in ASD [48] by making the estimation task an unspeeded binary button press (i.e., up or down) that additionally had to be ‘confirmed’ via another button press (see Methods). Likewise, scrutiny of response time distributions did not suggest different strategies across experimental groups. Further, even if there were differences in post-encoding processes, these differing strategies (part of the decoding process) trade off biases and variances, and thus we would regardless recuperate an appropriate estimate of encoding (see* ***Figure 1*** *and* ***Supplementary Note****). Nevertheless, to ascertain that potential stimuli-independent variability or post-perceptual factors do not deter from the current conclusions we ran additional simulations*

The analysis has been added to the supplementary materials in the following manner:

***Figure S5:*** *Histogram of response time (RT, i.e., time it took for subjects to rotate the response Gabor patch to match the target orientation). The overall shape of the distributions is qualitatively similar. There is a significant albeit small difference in the median RT between the control and ASD group across all three blocks (woFB, wFB1 and wFB2). Furthermore, there is a significant decrease in RT for both the control and ASD group with feedback as the experiment progresses.*

3. I am also skeptical of the suggestion that controls' priors are changed by exposure to stimuli with a uniform distribution of orientation. If that were to be the case, exposure alone should have sufficed; feedback should not have been necessary. Since anisotropies in orientation encoding are believed to arise from mere exposure to a world with increased representation of cardinal directions, why should the modification of these priors require feedback?

We thank the reviewer for this comment and the opportunity to clarify. We did not intend to claim that exposure alone modified encoding (at least not in the timeframe of the current experiment). We have thoroughly revised the text making sure there is no claim that exposure alone caused learning. Instead, we present participants with an artificial distribution (i.e., uniform), as a goal to attain; performance will be best when the allocation of encoding resources matches this new stimulus distribution. This new allocation of sensory resources is then to be reached during the experiment by incorporating perceptual feedback.

Now, it is likely that modifying the allocation of sensory resources in principle does not require feedback, if exposure is long enough. However, given that we were working with a clinical population, we provided feedback to accelerate the re-allocation of encoding resources. And indeed, examining the woFB block with a sliding window analysis suggests that if anything, participants became worse, and not better over the course of the first block of trials (with exposure alone and no feedback; **Figure S6**). Thus, our a-priori choice of including feedback blocks was warranted, driving the effects reported in the manuscript. The analysis showing that exposure alone during the woFB block in fact *decreased* performance in both the control and in individuals with Autism, and that feedback *increased* performance in both controls and (to a smaller degree) in individuals with Autism has been incorporated in the Supplementary Materials.

***Figure S6:*** *We conducted a sliding-window analysis in an attempt to reveal the effect of learning within each block (woFB, wFB1 and wFB2) in terms of changes in:* ***A)*** *average magnitude of the bias,* ***B)*** *standard deviation of the response,* ***C)*** *shape of the FI, and* ***D)*** *magnitude of the FI λ. Our analysis suggested that, within the context of our experiment, feedback seems essential for learning. In fact, within the first block (woFB), the bias and variance of both groups seem to be increasing as opposed to decreasing.*

Note that the fact that feedback drove the increased performance in controls (and to a lesser degree the reallocation of sensory resources in ASD), does not contradict the reported differences in encoding (total encoding capacity, even during block 1, and inflexibility in the allocation of these resources), given that 1) our method is able to isolate sensory encoding regardless of potential differences in other factors (e.g., individual differences in subject’s prior and loss function; **Figure 1; *Supplementary Note)*** and 2) sensory encoding is impacted by network-level properties, e.g., neuromodulators impacting neural gain, noise correlation, etc.

We have vastly re-written the manuscript in order to clarify these important points. The text reads:

Introduction:

*First, we had control and ASD individuals estimate orientations in the absence of feedback, in order to index their baseline encoding capacity. Then, to parallel observations emphasizing the slow updating of Bayesian priors in ASD [10, 11] and establish whether this phenotype would extend to sensory encoding, we presented participants with an experimental distribution of orientations (i.e., uniform) that differs from the natural (i.e., peaking at cardinal orientations) and performance feedback. Feedback was given in order to accelerate any putative re-allocation of encoding resources, from the natural to the artificial one.*

Results:

*We asked participants to perform a visual orientation estimation task, first without feedback, and then in the presence of trial-by-trial feedback in an attempt to further induce learning and updating of sensory encoding.*

Discussion*:*

*First, in the current experiment we provided participants with feedback on their performance in latter blocks of trials. This was done to accelerate learning of the novel statistical distribution of orientations and thus to enable re-allocation of encoding resources. And indeed, scrutiny within the woFB block suggests that no learning occurred – neither in control nor ASD individuals – in the absence of feedback (****Figure S6****), at least in the timeframe of our experiment. Thus, in the context of this experiment, seemingly feedback was necessary in reshaping encoding resources. This speculation goes hand-in-hand with our conjectures regarding the neural implementation of the current effect – likely being a network-level effect on the (feedforward) sensory representations in early visual areas (see [50] for an argument that the distribution of tuning functions in V1 underlies the oblique effect). In follow up experiments it will be interesting to repeat the current paradigm while considerably expanding the exposure time to the novel orientation distributions and without presenting feedback, in order to further understand if and how the updating of sensory encoding differs with mere exposure and with feedback.*

Similarly, we do not believe that the observed differences between ASD and control reflect a general inability to learn or incorporate feedback in the ASD population, as we have included in the discussion section:

*Related to the re-allocation of sensory resources, studies on perceptual learning have identified a multitude of processes that may contribute to learning, even in simple tasks. These include changes in low-level representations, changes in attention, decision rules, and/or the assimilation of feedback [40, 41]. Thus, one may wonder whether the observed inflexibility of sensory encoding in ASD reflects a more global learning deficit. We do not believe so, for the following reasons. First, the total amount of encoding resources differed between ASD and control already at the outset of the experiment, and, as stated above, this quantity correlated with the degree of re-allocation of encoding resources with feedback. Thus, if the inflexibility of encoding was expressing a global deficit in incorporating feedback, one would have to account for its correlation with total encoding resources at outset. Second, individuals with ASD did learn. Their allocation of encoding resources drifted from the natural toward the experimental distribution, particularly in wFB2 (****Figure S6****), just not as readily as in controls. More importantly, their response times decreased over the course of the experiment, and this effect was in fact larger in ASD than controls (****Figure S5****). Similarly, Harris and colleagues (2015) [42] have reported normal perceptual learning in individuals with ASD, in terms of improvement in discrimination threshold, as long as training and testing samples completely overlap (as was the case in the current experiment). It is only when probing for generalization in perceptual learning (i.e., from one retinotopic location to another) that poor performance is observed in ASD [42], again demonstrating a more specific, rather than a general, form of learning deficit.*

4. On a minor note, the classic oblique effect is associated with high orientation discrimination accuracy for horizontal and vertical orientations ('cardinal' directions) and least for obliques. In the data reported here, cardinal as well as oblique directions elicit high accuracies. What might account for this discrepancy? Might it be an outcome of the memory component of the task used here, with the four orientations being recalled symbolically ('horizontal', 'vertical', 'left diagonal', 'right diagonal')?

We thank the reviewer for this question and the opportunity to clarify. It turns out that the relationship between estimation bias and variance (measured here), and discrimination sensitivity (the reviewer’s question) is rather subtle. In fact, it is the (square-root of) Fisher information that directly determines discrimination sensitivity. In our experiment we observe that cardinal directions are associated with high FI (**Figure 4A**), thus suggesting that discrimination thresholds would be low at vertical and horizontal axis, as the reviewer indicates. The detailed derivation has been previously reported in the literature (Seriès, Stocker & Simoncelli, 2009; Wei & Stocker, 2017).

Briefly, assume the average internal estimate $\hat{x}$ of the stimulus $x$ is of the form $\hat{x}(x)=x+b(x)$, where $b(x)$ represents the bias, with an estimation variance of $\sigma^{2}$. Say we would like to discriminate between $x$ and $x+\delta x$. We can compute $d'=|\hat{x}(x)-\hat{x}(x+\delta x)| / \sigma$. Perform a linear approximation of $\hat{x}$ (for small $\delta x$, this is valid since we are interested in discrimination), we get $d'=|1+b'(x)| / \sigma$ which is the exact definition of the (square-root of) Fisher information. In other words, when the magnitude of bias (i.e., the $|1+b'(x)|$ term) is changing rapidly as is the case in orientation estimation, the relationship between discirmination sensitivity and variance is not straightforward. On the other hand, when the bias is close to a constant, fisher information, discrimination threshold, and (inverse of) variance coincide with each other quite nicely (as an example, see **Figure 1E**, Decoder 2).

We added the following sentence to clarify this issue and also provide references for readers interested in the relevant details.

*Results:*

*Using the Cramer-Rao bound (****Eq. 1****) we found that in both control and ASD individuals, FI peaked at cardinal orientations (****Figure 4A****). That is, initially the shape of FI (i.e., normalized* $\sqrt{I_{F}(\theta)}$*, see Methods,* ***Eq. 3****) in both groups qualitatively matched the previously measured distribution of orientations in natural images [21, 22]. Additionally, since FI directly determines discrimination thresholds [16, 32], the observed shape of FI also corroborates a heightened sensitivity to cardinal orientations [18] in both control and individuals with ASD.*

Overall, while I am sympathetic to the conceptual point the manuscript is attempting to make, I am not entirely convinced that the empirical data are adequately clear in the interpretations they allow.

We thank the reviewer for the constructive comments that have greatly improved the manuscript and particularly refined the interpretation of the results.

**Reviewer #2:**

In this ambitious paper, the authors attempt to take advantage of the so-called "oblique effect" to estimate aberrant sensory encoding in ASD. This is a really interesting and clever approach, but I have several concerns that significantly impact my enthusiasm for the manuscript in its current form. These are enumerated below.

1. Authors make the case that encoding, and decoding can be isolated behaviorally. However, the fullness of pathophysiology may come from the pairing between aberrant sensory encoding and some decoding scheme.

Our point here is that sensory encoding, at least within the context of grating orientation estimation, is different in ASD and control individuals. Many others (cited in text) have previously emphasized anomalies in decoding schemes. We do not claim that those previous reports are incorrect, we simply emphasize the less explored side of the equation, and do so via an information theoretic approach that allows to specifically examine sensory encoding regardless of the decoding scheme that may be employed. Given the previous evidence suggesting anomalies in decoding and the current evidence emphasizing deficits in sensory encoding, the reviewer is likely correct in suggesting that in the future we may have to consider both encoding and decoding to more fully account for the pathology present in ASD. We do wish to emphasize, however, that one of the main contributions of our current work is in applying to ASD a data analysis methodology that leverages a simple and lawful relationship (i.e., Cramer-Rao Lower Bound) to isolate and fully characterize encoding, regardless of potential differences in decoding (see the updated **Figure 1, D-F**).

We have amended the text in the following manner in order to more explicitly acknowledge that the current emphasis on sensory encoding is parallel to and does not detract from the characterization of sensory decoding in ASD (in fact it calls to the joint scrutiny of these).

Abstract:

*These findings highlight substantial deficits in sensory encoding – independent from and potentially in addition to deficits in decoding - in individuals with ASD, particularly when relating to these patients’ ability to adapt to novel input statistics.*

Introduction:

*Given the array of potential decoders the brain may employ and the various ways in which these may malfunction in ASD [8], here we were instead interested in identifying potential deficits in the sensory encoding, and the updating of this encoding with sensory exposure and feedback, in patients with ASD irrespective of differences in decoding. This exclusive emphasis on sensory encoding is seldom, if ever, explored within the ASD literature and does not detract from reported anomalies in sensory decoding [2-9], it simply adds to it.*

Discussion:

*Third, here we have exclusively focused on sensory encoding in an effort to separately understand each piece leading to perception (i.e., encoding, recoding, decoding). However, comprehensively understanding sensory processing in ASD will ultimately necessitate a broader framework including encoding, prior beliefs, and loss (utility) functions, among others. In the context of oblique effect this will require a non-trivial form of encoding [23], orientation prior [21], and loss function [52]. Thus, instead of “reverse-engineering” all these components together [53, 54], which would require a set of additional assumptions, here we took a data-driven approach. Our method, while not a complete model, can isolate and directly characterize the encoding process while being agnostic to potential differences in the decoding process and free of parametric restrictions about the shape of, for example, priors and likelihoods (see* ***Supplementary Note*** *for an extended discussion). As stated above, this is not to say that there are no differences in sensory decoding between the control and ASD group, but to simply emphasize anomalies in encoding. Future work will be able to build on this foundation to extend the comparison of sensory processing in ASD and control to a full model.*

2. Relatedly, others have demonstrated high-precision likelihoods in ASD. Why would this arise in the context of low-fidelity sensory encoding? Wouldn't one expect the opposite? Or is it the authors' contention that these two deficits are unrelated? This should be explained more fully in the Discussion.

At first glance this question is straightforward; yes, one would expect low-fidelity sensory encoding to result in broader, as opposed to narrower likelihoods. And as the reviewer states, there is literature suggesting sharper likelihoods in ASD (e.g., Brock, 2012; Karvelis et al., 2018). Although there is literature to the contrary as well (e.g., Park et al., 2017; Noel et al., 2020). However, we wish to highlight some subtleties. In principle, we could have an internal model of our senses that dictates a shift in mean or change in variance between these two distributions (as when wearing prisms, for example). Thus, while related, the exact relationship between likelihood functions and sensory encoding can vary and is one we do not fully understand. Second, and perhaps more relevant here, these abstract notions of a likelihood distribution must operate within the hierarchical architecture of the brain and thus it is possible that increased precision at one level of the hierarchy expresses as reduced precision at the next.

As suggested by the reviewer, we modify the *Discussion* in order to acknowledge the fact that some have observed sharper likelihood functions and failed to detect increased variability. The discussion reads;

*Lastly, we must mention that in a standard Bayesian framework, the lower encoding capacity of ASD would likely translate to broader likelihood functions, or underweighting of sensory evidence. While some prior work [29, 30, 36, 37] indeed suggest less precise sensory representation in ASD, others [55-58] have not replicated these observations. Butler et al., (2017) [58], for example, have shown no difference in the reliability of visual and somatosensory evoked activity during passive observation. Furthermore, some [3, 54] have even argued for more, and not less, precise sensory likelihoods in ASD. In turn, future work will have to charter the domain generality of deficits and/or improvements in sensory encoding in ASD. More broadly, we suggest caution must be taken when comparing “likelihood functions” in an encoding-decoding framework across studies, given that these represent an abstract and aggregated property of a complex and multi-layered hierarchical neural process, and could be strongly determined by factors such as stimulus type and task context.*

3. Bias and variance (Fig 1), observed behaviorally, are taken to be direct read-outs of the percept resulting from decoding. But of course, this cannot be—a response is generated somehow, and a response model must be specified if behavioral responses are used to estimate the quantities included in the model. What is this response model? If there is none, why not?

The reviewer is correct, in building a full model that is to *generate* putative responses, we would have to specify a response model - how posteriors are converted to a specific action. However, here there is no modeling in that sense, there are no degrees of freedom. Instead, we are simply applying the Cramer-Rao lower bound to the observed data (biases and variance) in order to compute Fisher Information. The approach is entirely data-driven and does not assume that bias and variance are *direct* readouts of an encoding-decoding process, but does leverage the lawful relationship between bias and variance (bias/variance trade-off) to determine encoding capacity. Of course, each participant may have their own response model. But this idiosyncratic loss-function is part of the decoding process. And as shown in **Figure 1** (**Figure S1** in the original submission), while different decoders would generate a different pattern of bias and variance, the Cramer-Rao lower bound would recuperate the appropriate encoding scheme in all cases (as jointly specified by both accuracy and precision).

We have modified the text in the following manner:

*Third, here we have exclusively focused on sensory encoding in an effort to separately understand each piece leading to perception (i.e., encoding, recoding, decoding). However, comprehensively understanding sensory processing in ASD will ultimately necessitate a broader framework including encoding, prior beliefs, and loss (utility) functions, among others. In the context of oblique effect this will require a non-trivial form of encoding [23], orientation prior [21], and loss function [52]. Thus, instead of “reverse-engineering” all these components together [53, 54], which would require a set of additional assumptions, here we took a data-driven approach. Our method, while not a complete model, can isolate and directly characterize the encoding process while being agnostic to potential differences in the decoding process and free of parametric restrictions about the shape of, for example, priors and likelihoods (see* ***Supplementary Note*** *for an extended discussion). As stated above, this is not to say that there are no differences in sensory decoding between the control and ASD group, but to simply emphasize anomalies in encoding. Future work will be able to build on this foundation to extend the comparison of sensory processing in ASD and control to a full model.*

4. The authors would do well to motivate the use of feedback blocks during the introduction. Feedback comes somewhat as a surprise when introduced in the Results section and the reader is unclear why this manipulation was introduced in the context of the overall study.

We thank the reviewer for highlighting this issue. We have amended the *Introduction* to emphasize that recent work within computational psychiatry has demonstrated the slow updating of priors while studying Bayesian decoding in ASD, and thus our interest here in paralleling those observations to the study of sensory encoding in ASD. We have modified the text in the following manner:

*Anomalies in this decoding process have been the main focus of recent computational studies of ASD (e.g., imbalance in the weighting of likelihood vs. priors; see citations above), with researchers recently also emphasizing a slow or inflexible updating of priors in ASD [10, 11].*

And:

*Given the array of potential decoders the brain may employ and the various ways in which these may malfunction in ASD [8], here we were instead interested in identifying potential deficits in the sensory encoding, and the updating of this encoding with sensory exposure and feedback, in patients with ASD irrespective of differences in decoding.*

And:

*To address this challenge, and to specifically test the hypothesis that sensory encoding and its updating is aberrant in ASD, we leverage a principled framework for data analysis emphasizing sensory encoding independently from decoding (****Figure 1B, C****).*

And:

*First, we had control and ASD individuals estimate orientations in the absence of feedback, in order to index their baseline encoding capacity. Then, to parallel observations emphasizing the slow updating of Bayesian priors in ASD [10, 11] and establish whether this phenotype would extend to sensory encoding, we presented participants with an experimental distribution of orientations (i.e., uniform) that differs from the natural (i.e., peaking at cardinal orientations) and performance feedback. Feedback was given to accelerate any putative re-allocation of encoding resources, from the natural to the artificial one.*

5. Related to 3, and especially in the context of feedback-based learning, it is not surprising that the authors show an improvement in FI while showing a decrease in bias. There are no other variables capable of explaining that difference, given the authors' model. This doesn't mean that the results are not due to simple response bias or any other post-encoding step of processing, but rather that the model, in its incompleteness, is incapable of accounting for any other reason for the behavioral results. The authors provide reasons why behavioral noise and decoding processes are unlikely to account for the results observed. But the only way to make this truly convincing is to test it formally by including a Bayesian decoding and response model with relevant parameters included.

We agree with the reviewer that there could well be other potential differences between the control and ASD group. We added the following to make this point explicitly:

*Third, here we have exclusively focused on sensory encoding, both as a contrast to prior work focusing exclusively on decoding and in an effort to separately understand each piece leading to perception (i.e., encoding, recoding, decoding). However, comprehensively understanding sensory processing in ASD will ultimately necessitate a broader framework including encoding, prior beliefs, and loss (utility) functions, among others. In the context of oblique effect this will require a non-trivial form of encoding [23], orientation prior [21], and loss function [49]. Thus, instead of “reverse-engineering” all these components [50, 51] here we took a data-driven approach. Our method, while not a complete model, can isolate and directly characterize the encoding process while being agnostic to potential differences in the decoding and free of parametric assumptions about the shape of, for example, priors and likelihoods (see* ***Supplementary Note*** *for an extended discussion). As stated above, this is not to say that there are no differences in sensory decoding between the control and ASD group, but to simply emphasize anomalies in encoding. Future work will be able to build on this foundation to extend the comparison of sensory processing in ASD and control to a full model.*

However, instead of “forcing” the improvement to be explained by encoding, our method does the exact opposite. We allow for any possible combinations of changes in prior and loss function (i.e., response strategy). Our analysis is agnostic to these potential differences, as long as the resulting decoder still achieves the Cramer-Rao Lower Bound. And again, this is a single assumption that is made by all decoders in neuroscience; Bayesian, MLE, linear classifiers, etc. If we were to build a “full model”, we would necessarily make many more assumptions (for example, regarding the shape of likelihoods and priors). Here, we instead use a lawful relation between variance and bias to *measure* the encoding process. Namely, there is no actual model being employed here, nor model parameters. We are simply leveraging a lawful relationship to calculate FI. This latter measure, will appropriate index sensory encoding, even in the face of differing decoders, given that those decoders would trade off variance and bias. We have modified **Figure 1** to now incorporate the simulations demonstrating that different decoders would lead to different patterns of bias and variance, but always giving the correct underlying encoding scheme.

***Figure 1. Theoretical and Conceptual Framework. A)*** *Perception can be described as an encoding-decoding process. Stimulus is first encoded in the noisy and resource limited sensory representation* $m$*. An estimate* $\hat{\theta}$ *is generated given* $m$ *based on the decoding process. As an experimenter, we can only measure overt responses; characterized as a subject’s bias (*$\mu\left( \hat{\theta} \right)- \theta$*; difference between the average estimate and real stimulus value) and variance* $\sigma^{2}\left( \theta\right)$ *in their responses.* ***B)*** *The fidelity of encoding in healthy subjects is generally anisotropic due to uneven allocation of sensory resources determined by environmental statistics. For example, as shown here, the non-linear transformation between stimulus values and a neural space (solid red line) with homogenous noise results in higher uncertainty for orientations around the oblique than those around the cardinal. This can be characterized by a non-uniform profile of Fisher information* $I_{F}\left( \theta\right)$ *(solid green line). In our experiment, we imposed an artificial, uniform distribution of orientations, different from that of natural environment, which allows us to study whether and how both groups update their encoding under changing stimulus statistics.* ***C)*** *Cramer-Rao Lower Bound specifies the lawful relationship between bias* $b\left( \theta\right)$*, variance* $\sigma^{2}\left( \theta\right)$*, and encoding accuracy, i.e., Fisher Information* $I_{F}\left( \theta\right)$*, regardless of the decoding scheme. This allows us to directly characterize encoding, while remaining agnostic about the details of the decoding process (also see Supplementary Text).* ***D)*** *To demonstrate validity of our approach, we simulate an observer with anisotropic encoding process,* $p\left( m | \theta\right)$*, with a peak FI at 90°.* ***E)*** *As an example, we construct three arbitrary decoders (red, black, and blue), yielding very distinct pattern of estimation biases and variances, yet they all attain the Cramer-Rao Lower Bound.* ***F)*** *Applying the inequality, we estimate the (lower bound of) FI, which appropriately recovers the identical, true underlying pattern of FI in the encoding regardless of idiosyncrasies in the decoding process.*

Further, we have amended the text in the following manner:

*In other words, by measuring bias (*$b)$ *and variance (*$\sigma^{2})$ *in estimates* $\left( \hat{\theta} \right)$ *of* $\theta$*, we can determine a lower bound on FI, i.e., the accuracy with which the stimulus variable is represented. Equating this bound with a direct index of sensory encoding (i.e., in effect replacing the greater than or equal sign with an equality in* ***Eq. 1****) requires a single assumption - that the translation from sensory representations to behavioral outputs was uncorrupted by stimulus-dependent noise. Note that this assumption is (often implicitly) made by virtually all decoders in perceptual science (e.g., Bayesian decoders, MLE), including neural decoders (e.g., linear classifiers, population vector, etc.). The exact form of the decoder (i.e., idiosyncrasies in the mapping from sensory estimates to motor outputs) does not matter beyond this single assumption, given that any decoder will trade off bias and variance. We further motivate and demonstrate the validity of our approach via both a numerical simulation (****Figure 1)*** *and an analytical derivation (see* ***Supplementary Note****) showing that: 1) the same encoding process (****Figure 1D****) can produce very distinct patterns of estimation bias and variance given different decoders (****Figure 1E****), yet critically, 2) with the Cramer-Rao Lower Bound, we are able to precisely recover the encoding scheme in terms of FI, regardless of the differences in decoding (****Figure 1F****).*

As an example, hypothetically say *without* any changes in encoding, subjects achieved a reduction in bias by changing their response strategy. Due to the inherent relationship between FI, bias, and variance, the estimation variance must change in a way that cancels out the change in bias, so when we compute the FI, we will find no changes (see the analytical derivation in the **Supplementary Note** for an explicit example showing how bias and variance can trade-off given a fixed encoding.

Nevertheless, one may wonder how a violation of our assumption (i.e., decoder achieves the lower bound) can potentially impact our analysis and interpretations. Thus, we have extended the discussion with additional simulations (**Figure S7** and **S8**) showing that the additional variability is either unlikely, or does not influence the conclusion of our analysis. This text reads as follows:

*Second, one may wonder about the potential impact of additional stimulus-independent variability introduced by factors following encoding – such as an inefficient decoder (i.e., a decoder that produces larger variance than that predicted by the Cramer-Rao lower bound), or post-perceptual factors such as motor noise. We attempted to experimentally minimize the potential impact of motor noise in ASD [51] by making the estimation task an unspeeded binary button press (i.e., up or down) that additionally had to be ‘confirmed’ via another button press (see Methods). Likewise, scrutiny of response time distributions did not suggest different strategies across experimental groups. Further, even if there were differences in post-encoding processes, these differing strategies (part of the decoding process) trade off biases and variances, and thus we would regardless recuperate an appropriate estimate of encoding (see* ***Figure 1*** *and* ***Supplementary Note****). Nevertheless, to further ascertain that potential stimuli-independent variability or post-perceptual factors do not deter from the current conclusions we ran additional simulations. We show that turning the decoder inefficient by adding homogeneous late noise will both decrease the total amount and flatten the pattern of extracted FI (****Figure S7****). This is incompatible with the observed results in the following aspects. First, our empirical data suggest exposure to an artificial stimulus distribution coupled with feedback increases total FI (i.e., the λ parameter) while flattening the pattern of FI (i.e., the* $\omega$ *parameter). Thus, if the increase in total FI with feedback in the control group were to be explained by an increase in the efficiency in the decoder or a decrease in motor variability, the pattern of FI ought to be sharpened, which is the exact opposite of what we observed in the data. Similarly, if we were to explain the lack of change in total FI in the ASD group with increase in additional noise, the pattern of FI would have to go through even less learning (****Figure S8A, B****). Lastly, if we were to explain the initial difference in total FI (i.e., before feedback) between ASD and controls with additional stimulus-independent variability in the ASD group, then the “true” (i.e., uncorrupted by late noise) FI pattern in ASD would have to be more extreme (i.e., sharper at cardinal orientations) than what is specified by the stimulus distribution in the natural environment (see* ***Figure S8C, D****). While we cannot completely rule out this latter possibility, we consider that it is most parsimonious that both control and ASD groups do indeed start with a pattern of FI that is similarly adapted to the natural statistics of orientations, just as we observed in our experiment.*

**Figure** **S8** has been included in the supplement in the following manner (**Figure S7** was already featured in the original submission of the manuscript:

***Figure S8:*** *In a further attempt to understand the potential impact of post-encoding noise, we conducted model simulations with the counterfactual assumption that the abnormalities in λ (both total and change thereof) in the ASD group are fully explained by stimulus-independent noise.* ***A)*** *We simulated an artificial observer (black solid) for which its FI increased during wFB2, just as for controls. However, due to additional noise this observer appears (black dashed) to be unchanged, similar as the ASD group (red dashed).* ***B)*** *The* $\omega$ *values in the artificial observer (black solid) would have to undergo less learning than what was observed in ASD, such that that when it is corrupted by additional noise, it appears to have similar* $\omega$ *values as the ASD group (red dashed).* ***C)*** *Similarly, we simulated another artificial observer (black solid) for which its FI matched that of the controls during woFB (blue dashed), yet appears to be reduced (black dashed) to a similar level as the ASD group (red dashed) due to additional noise.* ***D)*** *The* $\omega$ *values in the artificial observer (black solid) would have to be even more extreme (FI peaking even stronger than what is determined by the natural distribution of orientations in the environment), such that when it is corrupted by additional noise, it appears to have similar* $\omega$ *values as the ASD group (red dashed). We consider this unlikely since it is most parsimonious that both control and ASD groups do indeed start with a pattern of FI that is similarly adapted to the natural statistics of orientations. Importantly, in both cases, the* $\omega$ *values in the simulated observer strongly support our main conclusion that the control group reallocate their encoding resources toward uniform distribution more so than the ASD group did.*

**Reviewer 3:**

This study investigates sensory encoding in autism (ASD). Over the past decade, there has been an increase in work looking at sensory abnormalities in autism, but we still lack a good understanding of how sensory, namely visual, processing is atypical in autism. This study focuses on visual encoding, a critical early step in visual processing. This question is addressed in the context of visual orientation discrimination and combines convincing behavioral data (I’d say remarkably convincing given what is typically seen in ASD work) and sophisticated computational modeling. The conclusion is that sensory encoding is severely impaired in ASD.

I find this to be an interesting and a noteworthy study. The behavioral data are, to repeat, convincing and certainly indicate atypical orientation processing in ASD. The modeling is well done, and while I’m not ready to fully “buy” the ultimate conclusions (details below), even if those conclusions turn out to be not entirely correct, this work will still be an important addition to our understanding of how sensory processing could be impaired in ASD.

We thank the reviewer for his overall positive feedback. The reviewer (also reviewers 1 and 2) brought to our attention a number of important points worth discussing (e.g., the potential context specificity of the current results, the existence of contradictory results in the literature), and as such we decided to almost entirely re-write the discussion (see below).

1A. There are two key behavioral findings: (1) the overall difference in performance in the first block and (2) poor learning/adaptation of the ASD group over blocks. Both findings need to be better integrated with the existing literature, both to better contextualize the present results and to consider alternative explanations. The authors do link the first result to other studies showing increased variability in ASD, but more is needed there. For example, a recent study that examined variability of visual orientation processing is not mentioned. Also, it is worth mentioning work that questions the “noise hypothesis” in ASD. Both references are below.

Park, W. J., Schauder, K. B., Zhang, R., Bennetto, L., & Tadin, D. (2017). High internal noise and poor external noise filtering characterize perception in autism spectrum disorder. Scientific reports, 7(1), 1-12.

Butler, J. S., Molholm, S., Andrade, G. N., & Foxe, J. J. (2017). An examination of the neural unreliability thesis of autism. Cerebral cortex, 27(1), 185-200.

In re-reading our previous *Discussion* in light of this comment, we decided to vastly re-write and re-structure the discussion. We now first enumerate the results, and then discuss each in turn, appropriately contextualizing them. These sections read as follows:

*By tracking perceptual estimates while (i) exposing observers to an artificial stimulus distribution (i.e., uniformly distributed orientations) and presenting them with performance feedback, (ii) directly extracting Fisher Information (FI) of sensory encoding from psychophysical data, and (iii) parameterizing the shape of FI as a mixture of the cardinal and uniform distributions of orientation stimulus, we have estimated the total sensory encoding capacity and the change in its allocation with feedback in individuals with ASD. This combined approach resulted in a number of empirical observations. First, the initial pattern of FI as a function of orientation strongly resembled the known natural distribution of orientations in the natural environment [21, 22] in both ASD and control individuals. Second, already in the first block of trials, the total encoding resources for the representation of gratings in ASD is about 2/3 that of their neurotypical counterparts. Third, exposure to an experimental distribution of orientations, coupled with feedback, resulted in the appropriate re-allocation and an overall increase in encoding resources of neurotypical individuals. This was not the case in individuals with ASD. Lastly, initial encoding capacity correlated with the degree of adaptation from the natural to experimental allocation of encoding resources.*

*The observation that encoding capacity differs from the outset between ASD and control individuals is in line with ‘neural unreliability thesis’ [27] and a number of recent reports that have suggested heightened variability in ASD, be it in relatively low-level visual psychophysics [36, 37], more naturalistic and continuous tasks [31], neurovascular [28, 29] or physiological responses [27]. Particularly noteworthy and relevant to the current report is a study by Park and colleagues [37], where researchers had control and ASD individuals discriminate visual orientations. Then, via psychophysical modeling [38, 39] they were able to ascertain that individuals with ASD had increased internal noise and a reduced capacity for external noise filtering than neurotypical controls. These results are also in line with accounts suggesting a reduced use of Bayesian priors [2] or an overweighting of sensory likelihoods vs. expectations in ASD [5]. Namely, these expectations in essence serve as low-pass filters reducing trial-to-trial variability, and thus a decreased use of Bayesian priors could engender greater variability.*

*The second major observation is that the re-allocation of sensory encoding resources is inflexible in ASD relative to neurotypical individuals. Given that within the efficient coding hypothesis the allocation of encoding resources may directly reflect subjects’ prior in the Bayesian framework [23], these results are similarly consistent with recent experimental findings suggesting that individuals with ASD may update their priors slowly [11], putatively due to over-estimating the volatility of the environment [10]. Further, here we suggest an explanation for the apparently inflexibility of priors in ASD. Namely, when resources are allocated primarily to represent statistically likely events, the representation of a given external environment may be best. However, it also limits the degree of resources that may be devoted to distinguishing between statistically unlikely alternatives [34, 35]. These statistically unlikely events, however, are the most informative when attempting to infer whether the environment has changed. There is an inherent trade-off between coding for representational fidelity and for inference of changing environments given limited encoding resources [34, 35]. Thus, the pattern of results suggesting reduced encoding resources in ASD may indicate that this deficit is at the root of aberrant perceptual flexibility in ASD [10, 11]. This speculation is supported by a correlation between the total amount of encoding resources a participant demonstrated in the initial block of the experiment, and the degree to which their sensory encoding had adapted at the end of the experiment.*

Then, we discuss a number of open questions; (1) the impact of feedback, and (2) the contradicting findings regarding increased vs. reduced or intact variability in ASD. These sections read as follows:

*First, in the current experiment we provided participants with feedback on their performance in latter blocks of trials. This was done to accelerate learning of the novel statistical distribution of orientations and thus to enable re-allocation of encoding resources. And indeed, scrutiny within the woFB block suggests that no learning occurred – neither in control nor ASD individuals – in the absence of feedback (****Figure S6****), at least in the timeframe of our experiment. Thus, in the context of this experiment, seemingly feedback was necessary in reshaping encoding resources. This speculation goes hand-in-hand with our conjectures regarding the neural implementation of the current effect – likely being a network-level effect on the (feedforward) sensory representations in early visual areas (see [50] for an argument that the distribution of tuning functions in V1 underlies the oblique effect). In follow up experiments it will be interesting to repeat the current paradigm while considerably expanding the exposure time to the novel orientation distributions and without presenting feedback, in order to further understand if and how the updating of sensory encoding differs with mere exposure and with feedback.*

And:

*Third, here we have exclusively focused on sensory encoding in an effort to separately understand each piece leading to perception (i.e., encoding, recoding, decoding). However, comprehensively understanding sensory processing in ASD will ultimately necessitate a broader framework including encoding, prior beliefs, and loss (utility) functions, among others. In the context of oblique effect this will require a non-trivial form of encoding [23], orientation prior [21], and loss function [52]. Thus, instead of “reverse-engineering” all these components together [53, 54], which would require a set of additional assumptions, here we took a data-driven approach. Our method, while not a complete model, can isolate and directly characterize the encoding process while being agnostic to potential differences in the decoding process and free of parametric restrictions about the shape of, for example, priors and likelihoods (see* ***Supplementary Note*** *for an extended discussion). As stated above, this is not to say that there are no differences in sensory decoding between the control and ASD group, but to simply emphasize anomalies in encoding. Future work will be able to build on this foundation to extend the comparison of sensory processing in ASD and control to a full model.*

*Lastly, we must mention that in a standard Bayesian framework, the lower encoding capacity of ASD would likely translate to broader likelihood functions, or underweighting of sensory evidence. While some prior work [29, 30, 36, 37] indeed suggest less precise sensory representation in ASD, others [55-58] have not replicated these observations. Butler et al., (2017) [58], for example, have shown no difference in the reliability of visual and somatosensory evoked activity during passive observation. Furthermore, some [3, 54] have even argued for more, and not less, precise sensory likelihoods in ASD. In turn, future work will have to charter the domain generality of deficits and/or improvements in sensory encoding in ASD. More broadly, we suggest caution must be taken when comparing “likelihood functions” in an encoding-decoding framework across studies, given that these represent an abstract and aggregated property of a complex and multi-layered hierarchical neural process, and could be strongly determined by factors such as stimulus type and task context.*

1B. In addition, the change in performance over blocks is discussed as learning, but no work on perceptual learning in ASD is mentioned. For example, the paper listed below showed that perceptual learning in ASD is atypical. This raises the question of whether the present results could be a result of a boarder deficit in learning?

Harris, H., Israeli, D., Minshew, N., Bonneh, Y., Heeger, D. J., Behrmann, M., & Sagi, D. (2015). Perceptual learning in autism: over-specificity and possible remedies. Nature neuroscience, 18(11), 1574-1576.

We thank the reviewer for this very interesting question. We have modified the text to discuss perceptual learning. The passage reads as follows:

*Related to the re-allocation of sensory resources, studies on perceptual learning have identified a multitude of processes that may contribute to learning, even in simple tasks. These include changes in low-level representations, changes in attention, decision rules, and/or the assimilation of feedback [40, 41]. Thus, one may wonder whether the observed inflexibility of sensory encoding in ASD reflects a more global learning deficit. We do not believe so, for the following reasons. First, the total amount of encoding resources differed between ASD and control already at the outset of the experiment, and, as stated above, this quantity correlated with the degree of re-allocation of encoding resources with feedback. Thus, if the inflexibility of encoding was expressing a global deficit in incorporating feedback, one would have to account for its correlation with total encoding resources at outset. Second, individuals with ASD did learn. Their allocation of encoding resources drifted from the natural toward the experimental distribution, particularly in wFB2 (****Figure S6****), just not as readily as in controls. More importantly, their response times decreased over the course of the experiment, and this effect was in fact larger in ASD than controls (****Figure S5****). Similarly, Harris and colleagues (2015) [42] have reported normal perceptual learning in individuals with ASD, in terms of improvement in discrimination threshold, as long as training and testing samples completely overlap (as was the case in the current experiment). It is only when probing for generalization in perceptual learning (i.e., from one retinotopic location to another) that poor performance is observed in ASD [42], again demonstrating a more specific, rather than a general, form of learning deficit.*

2. The first block does not use feedback, while the 2nd and the 3rd blocks do. That decision needs to be explained and justified, but it is not. Why was adding feedback after the first block important? After all, feedback is not necessary for us to learn stimulus statistics (though it likely speeds up the process). My worry is that this leaves open a possibility that how the two groups responded to this change in feedback could be, in part, what drives the difference. For example, what if individuals with ASD ignored feedback? (btw, this is a hypothetical, I don’t have a reason to believe this). What I’m looking for here is a better justification of why the feedback was used in this specific way and are there good reasons to believe that the results would generalize to other ways of deploying feedback (or no feedback at all). That is, I’m not asking for new data (certainly not during COVID times).

We have amended the text in order to introduce this aspect of the work earlier (i.e., examining if reported anomalies in the updating of priors in ASD would extend to sensory encoding) and justify the use of feedback. The text reads:

*Then, to parallel observations emphasizing the slow updating of Bayesian priors in ASD [10, 11] and establish whether this phenotype would extend to sensory encoding, we presented participants with an experimental distribution of orientations (i.e., uniform) that differs from the natural (i.e., peaking at cardinal orientations) and performance feedback. Feedback was given to accelerate any putative re-allocation of encoding resources, from the natural to the artificial one.*

And:

*First, in the current experiment we provided participants with feedback on their performance in latter blocks of trials. This was done to accelerate learning of the novel statistical distribution of orientations and thus to enable re-allocation of encoding resources. And indeed, scrutiny within the woFB block suggests that no learning occurred – neither in control nor ASD individuals – in the absence of feedback (****Figure S6****), at least in the timeframe of our experiment.*

More importantly, we have now also performed the main analyses of the report within a sliding window, in order to examine if exposure alone, without feedback, was capable of increasing performance. This analysis (below, and in **Figure S5**) suggested that the total pool of encoding resources did not increase (actually it did the opposite) over the course of the block without feedback (first block). Thus, we consider that the increase in the total pool of encoding resources and the re-allocation of these resources in control individuals was likely driven by feedback.

Importantly, we do not believe that individuals with Autism simply showed a generalized unwillingness to incorporate feedback, given that:

1) Total encoding resources were different already at the outset. And the magnitude of encoding resources at the outset correlated with the degree to which resources were re-allocated during the course of the experiment.
2) Individuals with ASD did learn. In fact, they modified their responses times *more* than controls. They also did show a reallocation of encoding resources (especially in the later block of the experiment), just not as readily as controls.
3) Perceptual learning experiments (e.g., Harris et al., 2015, *Nature Neuroscience*) show that basic perceptual learning is intact in ASD. They show anomalies in generalizing this learning, but the basic ability to learn is there.

We have amended the text in the following manner:

*Thus, one may wonder whether the observed inflexibility of sensory encoding in ASD reflects a more global learning deficit. We do not believe so, for the following reasons. First, the total amount of encoding resources differed between ASD and control already at the outset of the experiment, and, as stated above, this quantity correlated with the degree of re-allocation of encoding resources with feedback. Thus, if the inflexibility of encoding was expressing a global deficit in incorporating feedback, one would have to account for its correlation with total encoding resources at outset. Second, individuals with ASD did learn. Their allocation of encoding resources drifted from the natural toward the experimental distribution, particularly in wFB2 (****Figure S6****), just not as readily as in controls. More importantly, their response times decreased over the course of the experiment, and this effect was in fact larger in ASD than controls (****Figure S5****). Similarly, Harris and colleagues (2015) [42] have reported normal perceptual learning in individuals with ASD, in terms of improvement in discrimination threshold, as long as training and testing samples completely overlap (as was the case in the current experiment). It is only when probing for generalization in perceptual learning (i.e., from one retinotopic location to another) that poor performance is observed in ASD [42], again demonstrating a more specific, rather than a general, form of learning deficit.*

3. The AQ results (Fig 5) are entirely un-informative. The reported correlations seem to be mostly driven by the overall group differences, with little evidence of within-group correlation effects. For these conclusions to stand, what is needed is evidence that **within** the ASD group, these results are correlates with the symptom severity.

Moreover, AQ is a somewhat outdated measure that relies a lot on imperfect stereotypes of what autism is, and it is not used in the diagnosis process (rather it is best used as a measure of autism-like behaviors and thinking among the general population). However, it does look like ADOS was collected.

We have removed the correlations with symptomatology from the main text (the raw data are still available in **Figure S3** and **S4**). The correlation between the total amount of encoding resources during the first block of the experiment with the degree of change in their allocation holds when analyzing the ASD and control group separately. This analysis has been added to the text, which reads as follows:

*In line with the “adaptive coding” hypothesis [31, 32], we found a strong correlation between these variables (*$R^{2}$ *= 0.407, p < 10^-3^,* ***Figure 5A****). The correlation holds separately for the control and ASD groups (****Figure 5B****). Thus, the inflexibility in resource re-allocation observed in individuals with ASD may emanate from their limited encoding resources.*

***Figure 5. Correlation between Sensory Encoding Capacity and Flexibility in Encoding Resources Allocation. A)*** *The flatness of FI after feedback (y-axis, indicating incorporation of stimulus statistics from the experiment) correlates strongly with total encoding resources before feedback (x-axis). The blue dots indicate individual control subjects and red dots indicate individual ASD subjects.* ***B)*** *The same correlation holds within both the control and ASD groups.*

Minor points:

-- I’d take out “patients” from the title. Many individuals with autism do not consider themselves to be patients.

We thank the reviewer for alerting us about this. We have modified the title and removed the word “patients”.

-- In discussion (page 9), it is stated “This speculation will have to be confirmed in physiological studies.” It is not clear how. Are we talking about EEG and fMRI or animal models?

This section of the *Discussion* was removed.

-- Materials and Procedures: stimulus sizes need to be given in visual degrees and not centimeters.

This has been corrected. Thank you.
