# Supplementary material for: Individuals with autism spectrum disorder have altered visual encoding capacity
Source: PLoS Biol. 2021 May 12;19(5):e3001215. doi: 10.1371/journal.pbio.3001215 (PMC8143398; doi:10.1371/journal.pbio.3001215)
Supplement: Attachment — Submitted filename: PlosBio_Letter_to_reviewers.docx [file pbio.3001215.s019.docx]

Editor:

1) Thank you for adapting your title in response to reviewer 3's comment that "Many individuals with autism do not consider themselves to be patients". Along these lines, we think the rest of the manuscript should be edited to reflect this. We think language calling individuals with ASD 'patients' should be changed throughout the manuscript and abstract. Similarly, we think that instead of calling the control group 'healthy' you might use 'neurotypical'.

We have modified the manuscript (abstract, main text, figure captions, and supplementary materials) to avoid calling individuals with ASD “patients”. We have also re-named the control group ‘neurotypical’ and avoided terms such as ‘healthy’.

2) We would also like to suggest an edit to the title, which we think might help make it a bit more direct and clear. If you agree, we think the title could be changed to "People with autism spectrum disorder have altered visual encoding capacity".

We thank the editorial team for the suggested title and agree it can be more direct and clearer. We agree to the title change, with a slight modification, “Individuals” as opposed to “People”. Complete title: “*Individuals with Autism Spectrum Disorder Have Altered Visual Encoding Capacity”.*

3) Ethics request: Please indicate whether your protocol, approved by the Baylor College and Medicine IRB adhered to the Declaration of Helsinki or other national or international ethical guidelines.

Yes, it adhered to the Declaration of Helsinki. This has been added to the text in the following manner:

The Institutional Review Board at Baylor College and Medicine approved this study (protocol number H-29411) in adherence to the Declaration of Helsinki, and all participants gave their written informed consent and/or assent.
4) Ethics Request: Please include the ID number of the protocol approved by the Baylor College and Medicine IRB.

Protocol number has been added.

5) Data Request: Please provide, as a supplementary file, the summary statistics used to generate each graph presented in your study. Please make sure to provide a legend for this file, and to refer to it in your data availability statement and figure legends (including supplementary figure legends). For example, to each figure legend, you could add a statement saying “The data underlying this figure can be found in the supplementary file S1_data”.

We have included supplementary files requested, which contains all summary data together with our complete data analysis code to reproduce every figure in our manuscript. We have provided detailed instructions such that readers with MATLAB access should be able to easily re-run all of our analysis. These, including the raw data are also available through our GitHub repository: <https://github.com/cpc-lab-stocker/ASD_Encoding_2020>

The text has been modified to point readers to either the supplementary files, or the public Github repository.

Reviewer #1: The authors have thoughtfully responded to the concerns I had raised in my earlier review. I believe the manuscript is now suitable for publication.

We thank the reviewer for his or her positive feedback.

Reviewer #2: The authors have done an impressive job of responding to my comments comprehensively. I have no further comments.

We thank the reviewer for his or her positive feedback.

Reviewer #3 (Duje Tadin): I appreciate the authors' careful work on this revision. It addresses the concerns I had about this manuscript, namely concerns about links with other literature on sensory noise and on learning. I also appreciate the detailed consideration and additional analyses on the role of feedback, and how the authors responded to concerns about the AQ analysis.

We thank the reviewer for his or her positive feedback.

Thank you for your time and consideration,

Dora E. Angelaki and Alan A. Stocker (on behalf of the co-authors)
